# Effects of Symptoms of Attention-Deficit/Hyperactivity Disorder (ADHD) and Oppositional Defiant Disorder (ODD) on Academic Performance and Educational Attainment

**DOI:** 10.1007/s10578-023-01598-7

**Published:** 2023-09-01

**Authors:** Sampo Seppä, Anu-Helmi Halt, Tanja Nordström, Tuula Hurtig

**Affiliations:** 1 Research Unit of Clinical Medicine, University of Oulu, P.O. Box 5000, 90014 Oulu, Finland; 2 Department of Psychiatry, Oulu University Hospital, Oulu, Finland; 3 Medical Research Center Oulu, Oulu University Hospital, University of Oulu, Oulu, Finland; 4 Northern Finland Birth Cohorts, Arctic Biobank, Infrastructure for Population Studies, Faculty of Medicine, University of Oulu, Oulu, Finland; 5 Research Unit of Population Health, University of Oulu, Oulu, Finland; 6 Clinic of Child Psychiatry, Oulu University Hospital, University of Oulu, Oulu, Finland

**Keywords:** ADHD, ODD, Academic performance, Educational attainment

## Abstract

The aim of this longitudinal population-based cohort study was to examine the effects of ADHD and ODD symptoms in adolescence on academic performance at age 16, and on educational attainment by the age of 32. The population studied here was the Northern Finland Birth Cohort 1986 (NFBC1986). The participants were classified into four groups: those with symptoms of ADHD, ODD, ADHD + ODD, and a control group. Early academic performance at the age of 16 years was based on the Joint Application Register for Secondary Education, and eventual educational attainment was derived from the registers of Statistics Finland and included information recorded up to 2018. Although symptoms of pure ODD had a negative effect on academic performance at school relative to the control group, this effect was weaker than that of pure ADHD symptoms. The ADHD + ODD group, both males and females, had the greatest deficits of all in educational attainment in adulthood and failed to progress to an institution of higher education as often as the control group. Symptoms of ODD in adolescent females predicted educational attainment in adulthood that extended no further than the compulsory comprehensive school level. The results remained statistically significant after adjustment for the educational level of the parents of the subjects, family type, and any psychiatric disorders (other than ADHD or ODD). The findings provide valuable information on the pervasive effects of co-occurring symptoms of ADHD and ODD that persist into adulthood.

## Introduction

According to the criteria quoted in the 5th edition of the Diagnostic and Statistical Manual of Mental Disorders (DSM-5) attention-deficit/hyperactivity disorder (ADHD) is a common neurodevelopmental disorder with childhood onset [1]. Its symptoms include inattention, hyperactivity, and impulsiveness, and it is commonly understood as being characterized by symptoms that are both cognitive and behavioural [2]. Despite the childhood onset of ADHD, its symptoms often persist into adulthood, and it is generally recognized as having a lifelong prevalence [3]. A global prevalence of 5.3% was obtained for ADHD in community samples of children and adolescents from 35 countries [4], while meta-analyses have shown the estimated prevalence of ADHD in adults aged 19–45 to be 2.5% [5].

One condition that is frequently combined with ADHD is oppositional defiant disorder (ODD), which is classified in the spectrum of disruptive behaviour disorders (DBD) and has approximately 50–60% expression with ADHD in community-based samples [6, 7]. It has been suggested that approximately 90% of patients with lifetime ODD meet the criteria for at least one other lifetime disorder, so that 35% of those who have ODD, for example, also have ADHD [8]. ODD involves problems with the self-control of emotions and behaviour [9], and its estimated global prevalence is 3.3% [10]. According to DSM-5, the symptoms of ODD consist of angry or irritable mood, vindictiveness towards others and argumentative or defiant behaviour [1].

Symptoms of ADHD are associated with low academic performance [1, 11, 12]. Academic problems have been explained by cognitive problems such as working memory, which is the most seriously compromised cognitive domain [13]. Symptoms of ADHD are related to long-term adverse academic outcomes and struggles to complete higher degrees of educational attainment [14].

The relation between symptoms of ODD and academic performance is not clear in the literature, although symptoms of ODD are related to problems in school [15, 16]. A Taiwanese school-based national epidemiological study reported that children with ODD felt that they had problems in academic performance, attitude, and behaviour at school [16]. In an American epidemiological study, children with symptoms of CD/ODD had an increased risk of below average teacher-rated school performance (OR 2.6, 95% CI 1.2–5.6), but the odds for ADHD alone and ADHD + CD/ODD were both about twice those for CD/ODD alone [17]. Meanwhile, a clinical based study of 162 children in the US found that symptoms of ODD were not associated with poorer academic achievement as measured by composite reading and mathematics test scores after controlling for ADHD symptoms [18]. Likewise, a study of 3785 elementary school children failed to find a unique association between symptoms of ODD and an academic competence factor (reading, arithmetic, and writing skills) rated by parents [19]. ODD symptoms have been found to predict difficulties later in life [20, 21]. The results from the University of Victoria’s community-based study, for example, showed that symptoms of ODD occurring in adolescence predicted lower levels of educational attainment in adulthood for males and lower levels of occupational prestige for both males and females [21].

Since ODD typically co-occurs with ADHD, only a few studies have included a pure ODD group without comorbid ADHD symptoms, and very few have relied on community samples. The aim of this longitudinal population-based cohort study was to examine the effects of ADHD and ODD symptoms in adolescence on academic performance at age 16, and on educational attainment by the age of 32.

## Methods

The basic population for this study was the Northern Finland Birth Cohort of 1986 (NFBC1986), which consisted of 9432 live-born children, whose estimated date of birth fell between 1st July 1985 and 30th June 1986 and have been followed up prospectively since then [22, 23].

At the 16-year follow-up, parents were asked to evaluate the existence of ADHD and ODD symptoms in their offspring by means of a questionnaire sent to the parents of 9215 adolescents whose address had been known at 15 years of age. The parents of 6985 of these adolescents (75.8%) returned the questionnaire together with a statement of their informed consent.

### Exposure Variables; Symptoms of ADHD and ODD

ADHD symptoms were assessed on the Strengths and Weaknesses of ADHD symptoms and Normal-behaviours (SWAN) scale [24], which comprises 30 items. It has a wide rating scale measuring both above and below average performance, which helps with discriminating the severity of symptoms. Parents were asked to evaluate their offspring by comparing them with other adolescents of the same age group in terms of skills such as controlling anxiety, focusing attention, and inhibiting impulsive behavior during tasks that require prolonged mental effort and during daily activities.

We defined the ADHD symptoms using the first 18 items on the SWAN scale, corresponding to the 18 symptoms of ADHD in the DSM-IV-TR [25], i.e., extending from the question “Give close attention to detail and avoid careless mistakes” to “Enter into conversations and games (control interrupting/intruding)”. Answering options were: Far Below Average = 3, Below Average = 2, Somewhat Below Average = 1, Average = 0, Somewhat Above Average =  − 1, Above Average =  − 2, and Far Above Average =  − 3 [24].

Screening for symptoms of ODD employed a 7-point rating scale comparable to that found in the SWAN scale and used eight items corresponding to the symptom criteria listed in the DSM-IV-TR [25] to define the symptoms. The items were “Control temper”, “Avoid arguing with adults”, “Follow adult requests or rules (follow directions)”, “Avoid deliberately doing things that annoy others”, “Assume responsibility for mistakes or misbehaviour”, “Ignore annoyances of others”, “Control anger and resentment” and “Control spitefulness and vindictiveness”.

One missing item was allowed in each subscale, ADHD and ODD, and replaced with the mean of that subscale for the adolescent concerned, a procedure that has been used in earlier studies [26]. The 95th percentile of the distribution was used as the cut-off point for the problems. Four groups were created in this way: (1) ADHD symptoms, (2) ODD symptoms, (3) ADHD + ODD symptoms, and (4) controls, with neither ADHD nor ODD symptoms.

### Outcome Variables; Academic Performance and Highest Educational Attainment

The academic performance data were based on the Joint Application Register kept by the Finnish National Agency for Education, which is generated from the results of the 9th grade comprehensive school diploma at the age of 15–16 years, in which the subjects examined are Finnish, general subjects (including history, religious studies, and geography), mathematics, science (including biology, physics, and chemistry), the arts and physical education. Finnish comprehensive schools use the grading scale 10–4, of which 10–5 indicate a pass and 4 a fail. This relates to the US system in that grades of 10–9 are equivalent to A, 8 to B, 7 to C, 5–6 to D, and 4 to F.

The educational attainment data were collected from the register of Statistics Finland and included information extending up to 2018, when the subjects were 32 years old. The findings were classified into three classes: (1) comprehensive school (2) upper secondary school and (3) institution of higher education. Comprehensive school refers to grades 1–9 in the Finnish compulsory school system, upper secondary school means grades 10–12 or vocational school, and an institution of higher education refers to a university degree or a university of applied sciences i.e., polytechnic. The highest educational level achieved was the primary outcome measure aimed at here.

### Statistical Methods

The classified variables were analysed by means of contingency tables using the Pearson χ2 test, while continuous variables (means of school grades) were tested with the Anova analysis of variance, and where appropriate, using the Welch test. Post hoc tests were used for continuous variables as well, using the Tukey test, and the Games-Howell test where appropriate. Educational attainment was analysed with multinomial logistic regression.

When comparing the educational attainment, “upper secondary school” was set as a reference category in the multinomial logistic regression model in order to observe the “comprehensive school” and “institution of higher education” categories. The sexes were considered separately throughout. Unadjusted odds ratios were first calculated for the ADHD, ODD, and ADHD + ODD groups, and adjusted odds ratios were then calculated.

The model was adjusted for the educational attainment of the parents, family type, and any psychiatric disorders (other than ADHD or ODD). Psychiatric disorders were obtained from the Care Register for Health Care maintained by the Finnish Institute for Health and Welfare: the specialized care inpatient (until 2016) and outpatient treatments (1998–2016) and primary care outpatient treatments (2011–2016). If participants had only ADHD or ODD in the health notifications, they were not considered in connection with this variable. 314.0B and 314.1A were used as diagnostic criteria for ADHD and 313.8A for ODD in ICD9, while F90.0 was used as a diagnostic criterion for ADHD and F91.3 for ODD in ICD10. The number of subjects totally ignored in this connection was 13. The educational attainments of the parents were based on the questionnaire, whereas those highest of the participants were taken directly from the register. Both are classified in the same manner, however. All the statistical analyses were carried out using SPSS 25.0 (SPSS Inc., USA). All the tests were two-tailed, and all p-values less than 0.05 were considered as statistically significant.

## Results

### Characteristics of the Study Population

The characteristics of the population studied here are shown in Table 1, where it is seen that 66.4% of the adolescents with symptoms of ADHD were males, whereas 53.8% of those in the group with ODD symptoms were females (Table 1). In the control group, 20.0% of the parents of the subjects had an institution of higher education as their highest educational attainment, while the figure for the ADHD + ODD group was 10.4%. Regarding the family type of the subjects, 78.5% of the adolescents in the control group lived with both of their biological parents, whereas this was the case for 64.7% of the adolescents in the ADHD + ODD group. 44.1% of the subjects in the ADHD + ODD group had some other psychiatric disorder or disorders than ADHD or ODD in the health notifications, while this was the case for 33.8% in the ODD group, 30.6% in the ADHD group, and 19.1% in the control group.

**Table 1:** Descriptive statistics of the study population studied

	ADHD, n (%)	ODD, n (%)	ADHD + ODD, n (%)	Controls, n (%)	χ^2^(df), p-value	Post hoc test^a^
Sex of subjects					47.6 (3), *p* < 0.001	A&O/AO/C, AO&O/C
Males	241 (66.4)	67 (46.2)	117 (57.4)	2768 (48.8)		
Females	122 (33.6)	78 (53.8)	87 (42.6)	2909 (51.2)		
Total	363 (100)	145 (100)	204 (100)	5677 (100)		
Highest educational attainments of parents of the subjects					23.4 (6), *p* < 0.001	A&C, AO&O/C
Comprehensive school	95 (26.8)	36 (25.4)	46 (24.0)	1288 (23.2)		
Upper secondary school	215 (60.7)	78 (54.9)	126 (65.6)	3156 (56.8)		
Institution of higher education	44 (12.4)	28 (19.7)	20 (10.4)	1109 (20.0)		
Family type of the subjects					50.1 (3), *p* < 0.001	A&C, C&O/AO
Both parents	231 (67.3)	93 (66.4)	123 (64.7)	4297 (78.5)		
Others	112 (32.7)	47 (33.6)	67 (35.3)	1177 (21.5)		
Psychiatric disorders of the subjects^b^					112.7 (3), *p* < 0.001	A&AO/C, O&AO/C, AO&C
Yes	111 (30.6)	49 (33.8)	90 (44.1)	1085 (19.1)		
No	252 (69.4)	96 (66.2)	114 (55.9)	4592 (80.9)		

*A* ADHD, *O* ODD, *AO* ADHD + ODD, *C* controls

^a^Pairwise crosstabulations between the study groups; only significant differences (*p* < 0.05) are noted

^b^Other than ADHD or ODD

### Academic Performance at the Age of 16

Data on the academic performance of the males at age 15–16 are shown in Table 2. The control group had the highest grades in all subjects, the mean grade for the control group in general subjects for example being 7.61 (SD 1.10, p < 0.001). Also, the ODD group had higher grades than ADHD and ADHD + ODD groups, so that the mean grade for the ODD group in general subjects, for example, was 7.14 (SD 1.20, p < 0.001) whereas it was 6.63 in the ADHD group (SD 1.00, p < 0.001) and 6.65 in the ADHD + ODD group (SD 1.11, p < 0.001). No differences were found between the ADHD and the ADHD + ODD groups (Table 2).

**Table 2:** School grades of males in the ADHD, ODD, ADHD + ODD and control groups

	ADHD Mean (SD)	ODD Mean (SD)	ADHD + ODD Mean (SD)	Controls Mean (SD)	F-value (DF)	Anova/Welch	Post hoc test^a^
Finnish	6.51 (0.97)	6.90 (1.15)	6.50 (1.02)	7.42 (1.10)	73.0 (3)	*p* < 0.001	C > A/O/AO
General subjects	6.63 (1.00)	7.14 (1.20)	6.65 (1.11)	7.61 (1.10)	82.6 (3)	*p* < 0.001	C > O > A/AO
Mathematics	6.50 (1.21)	7.00 (1.33)	6.65 (1.27)	7.58 (1.37)	71.6 (3)	*p* < 0.001	C > A/O/AO, O > A
Science	6.49 (0.97)	7.14 (1.14)	6.51 (1.14)	7.56 (1.16)	107.6 (3)	*p* < 0.001	C > O > A/AO
The arts	7.29 (0.93)	7.73 (0.93)	7.48 (0.91)	7.87 (0.86)	33.1 (3)	*p* < 0.001	C > A/AO, O > A
Physical education	7.79 (1.15)	7.86 (1.20)	7.50 (1.28)	8.27 (1.02)	25.8 (3)	*p* < 0.001	C > A/O/AO

Finnish comprehensive schools use the grading scale 10–4, of which 10–5 indicate a pass and 4 a fail. This relates to the US system in that grades of 10–9 are equivalent to A, 8 to B, 7 to C, 5–6 to D, and 4 to F

*A* ADHD, *O* ODD, *AO* ADHD + ODD, *C* controls

^a^Only significant differences (*p* < 0.05) are noted

The performance data for the females at age 15–16 are shown in Table 3. The control group had the highest grades in all subjects, the mean grade for the control group in Finnish for example being 8.37 (SD 0.94, p < 0.001). Again, the ODD group managed better than the ADHD or ADHD + ODD group, so that where the mean grade for the ODD group in Finnish, for example was 8.03 (SD 0.95, p < 0.001), it was 7.60 in the ADHD group (SD 0.86, p < 0.001) and 7.41 in the ADHD + ODD group (SD 1.06, p < 0.001). Again, no differences were found between the ADHD and ADHD + ODD groups. In general, however, the females managed better than the males (Tables 3).

**Table 3:** School grades of females in the ADHD, ODD, ADHD + ODD and control groups

	ADHD Mean (SD)	ODD Mean (SD)	ADHD + ODD Mean (SD)	Controls Mean (SD)	F-value, (DF)	Anova/Welch	Post hoc test^a^
Finnish	7.60 (0.86)	8.03 (0.95)	7.41 (1.06)	8.37 (0.94)	52.4 (3)	*p* < 0.001	C > O > A/AO
General subjects	7.25 (0.93)	7.75 (1.09)	7.14 (0.94)	8.18 (1.05)	55.7 (3)	*p* < 0.001	C > O > A/AO
Mathematics	6.80 (1.17)	7.42 (1.36)	6.58 (1.24)	7.83 (1.30)	47.6 (3)	*p* < 0.001	C > O > A/AO
Science	6.90 (0.97)	7.52 (1.15)	6.79 (1.04)	7.94 (1.11)	61.0 (3)	*p* < 0.001	C > O > A/AO
Art	8.33 (0.72)	8.64 (0.73)	8.21 (0.74)	8.63 (0.75)	13.9 (3)	*p* < 0.001	C > A/AO, O > A/AO
Physical education	7.79 (1.14)	8.11 (1.13)	7.36 (1.19)	8.34 (0.96)	26.1 (3)	*p* < 0.001	C > A/AO, O > AO

Finnish comprehensive schools use the grading scale 10–4, of which 10–5 indicate a pass and 4 a fail. This relates to the US system in that grades of 10–9 are equivalent to A, 8 to B, 7 to C, 5–6 to D, and 4 to F

*A* ADHD, *O* ODD, *AO* ADHD + ODD, *C* controls

^a^Only significant differences (*p* < 0.05) are noted

### Educational Attainment by the Age of 32

The educational attainments of the various groups are shown in Table 4 and the multinomial logistic regression model for educational attainment at the age of 32 in the Table 5. Where 6.3% of the male participants in the control group had comprehensive school as their highest educational attainment, this was the case for 23.0% of those in the ADHD + ODD group. In the unadjusted model the OR was 2.82 with a 95% CI of 1.76–4.52, and when the model was adjusted for the educational attainments of the parents, family type, and psychiatric disorders other than ADHD or ODD the result stayed statistically significant (OR 2.03, 95% CI 1.18–3.48).

**Table 4:** Educational attainments at age 32 years

	Males				Females			
	ADHD n (%)	ODD n (%)	ADHD + ODD n (%)	Controls n (%)	ADHD n (%)	ODD n (%)	ADHD + ODD n (%)	Controls n (%)
Comprehensive school	27 (11.5)	7 (11.1)	26 (23.0)	170 (6.3)	10 (8.3)	6 (7.9)	9 (10.7)	62 (2.2)
Upper secondary school	175 (74.5)	36 (57.1)	76 (67.3)	1401 (51.9)	74 (61.7)	33 (43.4)	57 (67.9)	1060 (37.5)
Higher education	33 (14.0)	20 (31.7)	11 (9.7)	1129 (41.8)	36 (30.0)	37 (48.7)	18 (21.4)	1708 (60.4)

**Table 5:** Educational attainments at age 32 years, multinomial logistic regression

		Males				Females			
		Unadjusted		Adjusted		Unadjusted		Adjusted	
		OR	95% CI	OR	95% CI	OR	95% CI	OR	95% CI
Comprehensive school*	ADHD	1.27	0.82–1.97	1.16	0.72–1.85	**2.31**	**1.14–4.69**	1.87	0.85–4.10
	ODD	1.60	0.70–3.66	1.42	0.61–3.29	**3.11**	**1.26–7.70**	**2.82**	**1.12–7.13**
	ADHD + ODD	**2.82**	**1.76–4.52**	**2.03**	**1.18–3.48**	**2.70**	**1.28–5.70**	**2.34**	**1.05–5.23**
	Controls	–	–	–	–	–	–	–	–
Higher education*	ADHD	**0.23**	**0.16–0.34**	**0.25**	**0.17–0.38**	**0.30**	**0.20–0.45**	**0.37**	**0.24–0.57**
	ODD	0.69	0.40–1.20	0.65	0.36–1.20	0.70	0.43–1.12	0.81	0.49–1.34
	ADHD + ODD	**0.18**	**0.095–0.34**	**0.22**	**0.11–0.43**	**0.20**	**0.11–0.33**	**0.20**	**0.11–0.36**
	Controls	–	–	–	–	–	–	–	–

The model was adjusted for the educational attainments of the parents of the subjects, family type, and psychiatric disorders other than ADHD or ODD. The control group was used as a reference

*Statistically significant figures (p < 0.05) are in bold type

41.8% of the participants in the control group had an institution of higher education as their ultimate educational attainment, whereas this was the case for 14.0% of those in the ADHD group (adjusted OR 0.25, CI 0.17–0.38) and 9.7% of those in the ADHD + ODD group. The OR in the unadjusted model was 0.18 with a 95% CI of 0.095–0.34, and the result remained statistically significant when the model was adjusted (OR 0.22, 95% CI 0.11–0.43).

Among the females, 2.2% of the participants in the control group had comprehensive school as their highest educational attainment, while the figure for the ODD group was 7.9% (adjusted OR 2.82, 95% CI 1.12–7.13) and that for the ADHD + ODD group, 10.7%, in the unadjusted model with an OR of 2.70 with a 95% CI 1.28–5.70, a result that remained statistically significant in the adjusted model (OR 2.34, 95% CI 1.05–5.23).

60.4% of the female participants in the control group had an institution of higher education as their ultimate educational attainment, as opposed to 30.0% of those in the ADHD group (adjusted OR 0.37 with a 95% CI of 0.24–0.57) and 21.4% of those in the ADHD + ODD group, with an OR of 0.20 and a 95% CI of 0.11–0.33 in the unadjusted model, a result that remained statistically significant when the model was adjusted (OR 0.20, 95% CI 0.11–0.36). (Tables 4 and 5).

## Discussion

The findings that emerged from this large longitudinal birth cohort study showed that ODD symptoms in both the male and female adolescents predicted poorer academic performance than that observed in the controls. Although symptoms of pure ODD had a negative effect on academic performance relative to the control group, this effect was weaker than that of pure ADHD symptoms. No differences were found between the ADHD and the ADHD + ODD groups. In general, the association between ODD symptoms and poor academic performance is controversial. Several authors have failed to find any such correlation [18, 19, 27], while some have [17]. Furthermore, we found here that symptoms of ODD in adolescent females predicted educational attainment in adulthood that extended no further than the compulsory comprehensive school level, but this relation did not reach statistical significance in the males after adjustment for the educational attainments of the parents of the subjects, family type and psychiatric disorders other than ADHD or ODD.

Our results are consistent with previous findings and suggest that symptoms of ADHD are associated with poor academic performance. Attention problems during childhood have been shown to predict lower academic performance in adolescents [28], and symptoms of ADHD, especially those involving inattention, predicted lower reading, spelling, and mathematics scores in adolescence after controlling for intelligence [29]. The relationship between ADHD symptoms and academic achievement has been described as being mediated through classroom performance and homework management [30]. Our findings showed that the group with pure ADHD symptoms, both males and females, did not progress to an institution of higher education as often as in the control group.

The findings of our study suggested that the co-occurrence of ODD symptoms with ADHD symptoms in adolescence predicted the greatest deficits of all in educational attainment in adulthood for both males and females. This is consistent with the results of the study in which Drabick et al., suggested that symptoms of ODD synergistically aggravated problems of academic performance in children with symptoms of ADHD [31]. Liu et al., have likewise suggested that while symptoms of ADHD are associated with poor academic performance, co-occurrent ODD symptoms are associated with a more negative attitude towards schoolwork, disturbed social interaction, and increased behavioural problems [32]. These reasons may explain the fact that the highest educational attainment deficits in adulthood found in the present study were brought on in the long run by co-occurrent symptoms of ODD and ADHD. The fact that the results remained statistically significant in the adjusted model means that they provide valuable information on the pervasive effects of ODD symptoms co-occurring with symptoms of ADHD as the adolescent moves into adulthood. Moreover, we also managed to examine pure ADHD symptoms without comorbidities, obtaining findings that support those of previous studies and constitute further evidence for a strong relation between ADHD symptoms and poor academic performance.

### Strengths and Limitations

The strength of this study lies in the large, unselected population-based sample. ADHD symptoms were screened with the SWAN scale, which measures the full range of behaviour and not only pathological symptoms and signs of ADHD [33]. This serves well to emphasize information on different dimensions of population alterability [26]. ODD symptoms were screened with a comparable scale and were also observed in cases lacking the usual co-morbid ADHD symptoms, which is a further strength of this study. Information on academic performance was obtained from the registers of Statistics Finland, which means that the information is reliable, and the attrition rate was small.

One limitation of this study was that the ADHD and ODD symptoms were screened at the age of 15–16 years, when the participants were in the ninth grade. It is well known that symptoms of ADHD and ODD can be unduly emphasized in adolescents at puberty, but in the present case the symptoms were assessed by the parents rather than by the young people themselves. ADHD and ODD symptoms cannot be regarded as equal to diagnoses of ADHD and ODD, but when the whole spectrum of these symptoms is observed rather than diagnoses, the findings can still provide valid and clinically significant information at the population level.

## Summary

The findings that emerged from this large longitudinal birth cohort study showed that the co-occurrence of ODD symptoms with ADHD symptoms in adolescence predicted the greatest deficits of all in educational attainment in adulthood. Also, adolescents with ADHD symptoms alone, failed to progress to an institution of higher education as often as in the control group. Symptoms of ODD in adolescent females predicted educational attainment in adulthood that extended no further than the compulsory comprehensive school level. The results remained statistically significant after adjustment for the educational level of the parents of the subjects, family type, and any psychiatric disorders (other than ADHD or ODD). Understanding the factors behind academic underachievement can help us to prevent undesired outcomes. The role of parents and teachers is important, and early perception of problems with homework management and classroom performance can have significant long-term implications. The findings provide valuable information on the pervasive effects of co-occurring symptoms of ODD and ADHD that persist into adulthood.
